# 伴FGFR3基因突变初诊多发性骨髓瘤患者临床特征和预后分析

**DOI:** 10.3760/cma.j.issn.0253-2727.2023.12.004

**Published:** 2023-12

**Authors:** 娜 沈, 珏 张, 园 夏, 旭星 沈, 静 王, 媛媛 金, 闰 张, 建勇 李, 丽娟 陈

**Affiliations:** 南京医科大学第一附属医院，江苏省人民医院血液科，南京 210029 Department of Hematology, the First Affiliated Hospital of Nanjing Medical University, Jiangsu Province Hospital, Nanjing 210029, China

**Keywords:** 多发性骨髓瘤, 基因，FGFR3, t（4;14）, 二代测序, 基因突变, Multiple myeloma, Gene, FGFR3, t（4;14）, Next-generation sequencing, Gene mutation

## Abstract

**目的:**

探讨FGFR3基因突变对初诊多发性骨髓瘤（MM）患者临床特征及预后的影响。

**方法:**

回顾性分析2016年1月至2023年2月江苏省人民医院血液科诊治的198例初诊MM患者，所有患者采用二代测序技术检测FGFR3基因突变，以及胞质轻链免疫荧光结合荧光原位杂交技术检测t（4；14）核型，使用Log-rank检验和Cox风险比例回归模型分析FGFR3基因突变与临床特征及预后的相关性。

**结果:**

198例患者中，28例（14.1％）伴FGFR3突变。与无FGFR3突变的患者相比，FGFR3突变的患者初诊时白蛋白水平明显降低（*P*＝0.012），β_2_-微球蛋白水平明显升高（*P*＝0.014），t（4；14）阳性率增加（*P*<0.001），R-ISS分期Ⅲ期比例更高（*P*<0.001）。相较于无FGFR3突变患者，FGFR3突变患者具有更短的无进展生存（PFS）期（28个月对33个月，*P*＝0.024）和总生存（OS）期（54个月对未达到，*P*＝0.028）。联合FGFR3突变和 t（4；14）进行生存分析，FGFR3突变和（或）t（4；14）阳性患者较FGFR3突变且t（4；14）均阴性组患者PFS期（30个月对38个月，*P*＝0.012）和OS期（54个月对未达到，*P*＝0.017）均缩短。Cox比例风险回归分析显示，FGFR3突变是影响初诊MM患者PFS及OS的独立危险因素。

**结论:**

FGFR3基因突变与初诊MM患者预后不良显著相关。

## Abstract (en)

**Objective:**

This study aimed to investigate the influence of FGFR3 gene mutations on the clinical characteristics and prognosis of patients with newly diagnosed multiple myeloma (NDMM).

**Methods:**

A total of 198 patients with NDMM admitted to the Department of Hematology in Jiangsu Province Hospital between January 2016 and February 2023 were retrospectively analyzed. Next-generation sequencing and cytoplasmic light chain immunofluorescence with fluorescence in situ hybridization were performed for all patients. The prognostic significance of FGFR3 mutation and clinical features were analyzed using the Log-rank test and Cox proportional hazards model.

**Results:**

Among 198 patients, 28 carried the FGFR3 gene mutation. These patients had significantly lower serum albumin levels, higher β_2_-microglobulin levels, advanced Revised International Staging System stages, more frequent occurrence of t（4;14）, and shorter median progression-free survival（PFS）time（28 months *vs* 33 months, *P*＝0.024）and overall survival（OS）time（54 months *vs* undefined, *P*＝0.028）than patients without FGFR3 mutation. Additionally, patients carrying either FGFR3 mutation or t（4;14）had lower PFS（30 months *vs* 38 months, *P*＝0.012）and OS（54 months *vs* undefined, *P*＝0.017）than those without. The Cox proportional hazards model identified FGFR3 mutation as an independent risk factor for PFS and OS.

**Conclusion:**

FGFR3 gene mutation was an unfavorable independent prognostic predictor for NDMM.

多发性骨髓瘤（MM）是骨髓克隆性浆细胞异常增殖的恶性肿瘤，发病率居血液系统肿瘤第2位，具有遗传学背景复杂、基因突变数量多及预后显著异质性等特点[1]。近年来，随着分子生物学及遗传学检测技术的不断发展，发现了众多与MM高度相关的基因改变。约半数MM患者存在染色体14q32上的免疫球蛋白重链（immunoglobulin heavy chain，IgH）基因易位，最常见的伙伴基因位点主要有11q13、4p16、6q23、20q11等，其与染色体4p16上的纤维母细胞生长因子受体3（fibroblast growth factor receptor 3，FGFR3）或多发性骨髓瘤SET结构域蛋白（multiple myeloma SET domain protein，MMSET）基因易位时形成的t（4；14）是MM中公认的高危遗传学改变，在MM中的发生率约为15％[2]。研究发现，t（4；14）患者中70％出现FGFR3高表达，所有患者都有MMSET高表达[3]；因此，既往针对t（4；14）的深入研究常聚焦于MMSET，而对FGFR3的报道也主要针对其表达水平的变化，对FGFR3突变的分析相对较少，尤其是中国患者FGFR3突变的特征及预后价值鲜有报道。为此，我们采用二代测序（next-generation sequencing，NGS）技术对初诊MM患者的FGFR3突变进行分析，探讨FGFR3突变在中国MM患者中的临床意义。

## 病例与方法

1. 病例：纳入2016年1月至2023年2月在江苏省人民医院诊治的198例初诊MM患者，所有患者均完善NGS检测及胞质轻链免疫荧光结合荧光原位免疫杂交（cIg-FISH）检测，MM的诊断均符合《中国多发性骨髓瘤诊治指南（2015年修订）》的诊断标准[4]。收集患者初诊时的临床资料，包括年龄、性别、M蛋白类型、ISS分期、R-ISS分期、HGB、白蛋白、LDH、血肌酐、诱导治疗方案等。本研究经江苏省人民医院医学研究伦理委员会批准（批件号：2022-SR-448），并获得患者知情同意。

2. NGS技术：首先采用密度梯度离心分离初诊MM患者的骨髓单个核细胞，并通过CD138磁珠（Miltenyi公司）分选CD138阳性的MM细胞。采用QIAamp® Blood DNA Mini Kit试剂盒提取DNA，并使用NanoDrop检测DNA的浓度和纯度。委托上海源奇生物医药科技有限公司对DNA进行FGFR3测序。使用fastp软件对原始fastq测序文件进行质控，据COSMIC数据库中Cancer Gene Census列表对纳入分析的突变基因进行筛选。

3. 随访：通过电话以及查阅患者门诊和住院病历对患者进行随访，随访时间截至2023年5月31日，中位随访时间为34（4～79）个月。无进展生存（PFS）期定义为从确诊至疾病进展、复发或死亡的时间。总生存（OS）期定义为从确诊至任何因素导致的患者死亡或者随访终点的间隔时间。

4. 统计学处理：采用SPSS 20.0和GraphPad Prism 8.0软件进行统计学分析。计数资料的比较采用*χ*^2^检验；采用Kaplan-Meier曲线进行生存分析，生存数据的比较采用Log-rank检验；采用Cox风险比例回归模型分析预后影响因素。统计学方法均使用双侧检验，*P*<0.05为差异有统计学意义。

## 结果

1. 一般临床特征：198例患者中男115例、女83例，中位年龄63.5（29.0～86.0）岁。全部患者中40例（20.2％）存在t（4；14），28例（14.1％）存在FGFR3突变。进一步分析发现，FGFR3突变的患者中有22例（78.6％）伴t（4；14），提示两者发生的高度连锁型（图1）。

**图1:**
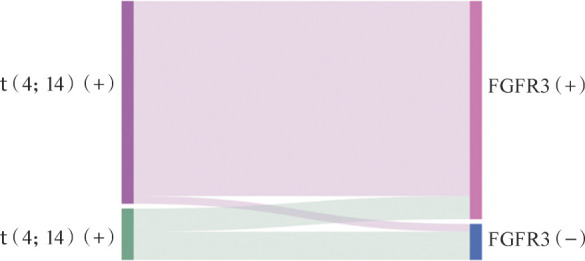
初诊多发性骨髓瘤患者t（4；14）及FGFR3突变发生情况比较

对患者初诊时临床特征的比较显示，两组患者的性别、年龄、M蛋白类型、ISS分期、HGB、LDH、血肌酐、血清钙、骨髓浆细胞比例等差异均无统计学意义（*P*值均>0.05）。与无FGFR3突变组患者相比，FGFR3突变的患者初诊时白蛋白水平明显降低（*P*＝0.012），β_2_-微球蛋白水平明显升高（*P*＝0.014），t（4；14）阳性率增加（*P*<0.001），R-ISS分期Ⅲ期比例更高（*P*<0.001）。对患者的诱导治疗方案进行比较，两组患者在使用蛋白酶体抑制剂或免疫调节剂为基础的诱导治疗方案与接受蛋白酶体抑制剂和免疫调节剂三药联合的诱导治疗方案差异无统计学意义（*P*>0.05），且两组患者行自体造血干细胞移植的比例差异亦无统计学意义（*P*>0.05）（表1）。

**表1:** FGFR3突变和无FGFR3突变的初诊多发性骨髓瘤患者临床特征比较［例（％）］

临床特征	FGFR3突变组（28例）	无FGFR3突变组（170例）	*P*值
男性	18（64.3）	97（57.1）	0.473
年龄[岁，M（范围）]	64（40～84）	63（29～86）	0.524
M蛋白类型			0.288
IgG	16（57.1）	88（51.8）	
IgA	9（32.1）	37（21.8）	
轻链型	2（7.1）	34（20.0）	
其他	1（3.6）	11（6.5）	
ISS分期			0.520
Ⅰ～Ⅱ期	12（42.9）	84（49.4）	
Ⅲ期	16（57.1）	86（50.6）	
R-ISS分期			<0.001
Ⅰ～Ⅱ期	12（42.9）	136（80.0）	
Ⅲ期	16（57.1）	34（20.0）	
HGB<100 g/L	15（53.6）	83（48.8）	0.641
白蛋白<35 g/L	24（85.7）	104（61.2）	0.012
LDH≥270 U/L	5（17.9）	26（15.3）	0.779
β_2_-微球蛋白≥5.5 mg/L	21（75.0）	85（50.0）	0.014
血肌酐≥177 µmol/L	11（39.3）	44（25.9）	0.142
血清钙>2.65 mmol/L	2（7.1）	23（13.5）	0.540
高危细胞遗传学异常			
gain（1q）	18（64.3）	93（54.7）	0.344
del（17p）	2（7.1）	19（11.2）	0.744
t（4；14）	22（78.6）	18（10.6）	<0.001
骨髓浆细胞比例≥30%	12（42.9）	59（34.7）	0.405
伴髓外病变	5（17.9）	35（20.6）	0.739
诱导治疗方案			0.625
PIs或IMiDs为基础的方案	18（66.7）	105（61.8）	
PIs+IMiDs的三药方案	9（33.3）	65（38.2）	
auto-HSCT治疗	4（14.3）	39（22.9）	0.303

注 auto-HSCT：自体造血干细胞移植；PIs：蛋白酶体抑制剂；IMiDs：免疫调节剂

2. FGFR3突变及t（4；14）对初诊MM患者的影响：198例MM患者中28例（14.1％）患者伴FGFR3突变，170例（85.9％）患者无FGFR3突变，FGFR3突变组与无FGFR3突变组患者的中位PFS期分别为28个月和33个月（*P*＝0.024），中位OS期分别为54个月和未达到（*P*＝0.028），FGFR3突变组的PFS和OS期均较无FGFR3突变组缩短，差异均有统计学意义。198例MM患者中t（4；14）阳性40例、t（4；14）阴性158例，与t（4；14）阴性的患者相比，t（4；14）阳性的患者PFS（30个月对34个月，*P*＝0.048）和OS期（43个月对未达到，*P*＝0.046）均缩短。

联合FGFR3突变和t（4；14）将198例患者分成两组：FGFR3突变（+）和（或）t（4；14）（+）组患者46例，FGFR3突变（−）且t（4；14）（−）组患者152例，FGFR3突变（+）和（或）t（4；14）（+）组患者较FGFR3突变（−）且t（4；14）（−）组患者PFS（30个月对38个月，*P*＝0.012）和OS期（54个月对未达到，*P*＝0.017）均缩短（图2）。

**图2:**
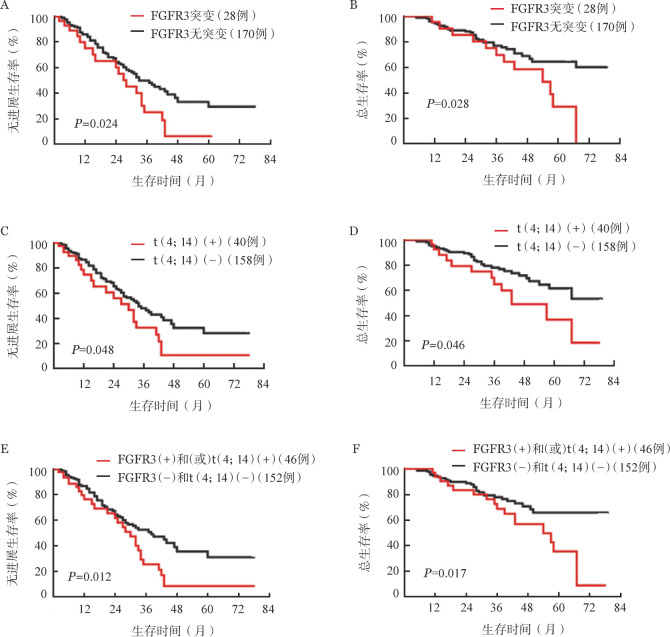
FGFR3突变及t（4；14）对初诊多发性骨髓瘤患者预后的影响 A 携带FGFR3突变对无进展生存期的影响； B 携带FGFR3突变对总生存期的影响； C 携带t（4；14）对无进展生存期的影响； D 携带t（4；14）对总生存期的影响； E 携带t（4；14）或FGFR3突变对无进展生存期的影响； F 携带t（4；14）或FGFR3突变对总生存期的影响

3. 初诊MM预后影响因素分析：对198例MM患者的预后进行单因素分析，纳入因素包括FGFR3突变（+）、性别、年龄、ISS分期、髓外病变、HGB、白蛋白、血肌酐、血清钙、LDH、gain（1q）、del（17p）、诱导治疗方案和auto-HSCT。随后，纳入单因素分析中*P*<0.05的参数，并对高度相关的参数进行筛选后进行多因素分析，结果显示，影响患者PFS的独立危险因素包括FGFR3突变（+）（*HR*＝0.57，95％*CI* 0.34～0.95，*P*＝0.032）、gain（1q）（*HR*＝1.69，95％ *CI* 1.08～2.64，*P*＝0.022）、del（17p）（*HR*＝1.89，95％*CI* 1.03～3.48，*P*＝0.039）和行auto-HSCT治疗（*HR*＝0.38，95％*CI* 0.21～0.67，*P*＝0.001），影响患者OS的独立危险因素包括FGFR3突变（+）（*P*＝0.035）、del（17p）（*P*＝0.006）及行auto-HSCT治疗（*P*＝0.004）（表2）。

**表2:** 影响多发性骨髓瘤患者生存的单因素和多因素回归分析

影响因素	无进展生存				总生存			
	单因素分析		多因素分析		单因素分析		多因素分析	
	*HR*（95%*CI*）	*P*值	*HR*（95%*CI*）	*P*值	*HR*（95%*CI*）	*P*值	*HR*（95%*CI*）	*P*值
FGFR3突变	0.56（0.34～0.93）	0.026	0.57（0.34～0.95）	0.032	0.50（0.26～0.94）	0.033	0.49（0.26～0.95）	0.035
男性	0.97（0.63～1.50）	0.893			0.85（0.47～1.53）	0.592		
年龄≥65岁	1.24（0.80～1.90）	0.335			1.86（1.04～3.31）	0.037	1.27（0.69～2.36）	0.447
ISS分期Ⅲ级	1.38（0.90～2.12）	0.138			1.34（0.76～2.39）	0.314		
髓外病变	1.40（0.86～2.27）	0.178			1.04（0.53～2.05）	0.908		
HGB<100 g/L	1.37（0.89～2.11）	0.154			1.36（0.76～2.43）	0.296		
白蛋白<35 g/L	1.04（0.67～1.61）	0.874			1.56（0.84～2.90）	0.157		
血肌酐≥177 µmol/L	1.39（0.89～2.18）	0.151			1.68（0.93～3.02）	0.085		
血清钙>2.65 mmol/L	1.52（0.76～3.05）	0.238			1.75（0.74～4.16）	0.204		
LDH≥270 U/L	1.06（0.59～1.92）	0.843			1.71（0.82～3.55）	0.149		
gain（1q）	1.77（1.14～2.75）	0.011	1.69（1.08～2.64）	0.022	1.72（0.95～3.11）	0.076		
del（17p）	1.87（1.04～3.39）	0.038	1.89（1.03～3.48）	0.039	2.49（1.16～5.37）	0.019	3.17（1.40～7.17）	0.006
PIs或IMiDs为基础的方案	0.64（0.38～1.08）	0.093			0.53（0.23～1.19）	0.123		
行auto-HSCT治疗	0.38（0.21～0.68）	0.001	0.38（0.21～0.67）	0.001	0.11（0.03～0.44）	0.002	0.12（0.03～0.52）	0.004

注 auto-HSCT：自体造血干细胞移植；PIs：蛋白酶体抑制剂；IMiDs：免疫调节剂

## 讨论

基因组不稳定是MM较为突出的生物学特征，根据测算MM细胞的每一百万个碱基中就有1.6个突变[5]。近年来，高通量测序技术的发展使得国内外学者对MM的突变特征有了更深入的认识，诸如TP53、KRAS、NRAS、FAM46C等已经得到广泛研究。IgH易位被认为是MM中的早期分子遗传学事件，其与非同源染色体各自断裂后相互交换部分基因片段，该过程可引起断裂点处部分基因的改变，还可因结构变化导致整个基因功能丧失，此外，易位后的基因可受新位点的上游调控元件影响，从而引起细胞的生物学特性改变；深入研究发现，癌基因与IgH易位后可受IgH的超级增强子调控进而出现转录失调，促进了MM的发生与发展，其中，FGFR3基因与IgH易位后就可因上述机制引起突变和（或）过表达[6]。

由于FGFR3是t（4；14）的主要受累基因，t（4；14）患者的FGFR3突变率显著增高[6]。早年的研究显示t（4；14）患者中仅有约5％检测到FGFR3突变，全部初诊MM患者中FGFR3突变率不足1％[7]–[8]。随后的NCRI Myeloma Ⅺ研究对初诊MM患者的突变谱分析显示，初诊MM患者FGFR3突变率为2.16％，亚组分析发现t（4；14）的患者中16.9％合并FGFR3突变，且均为非同义突变；该研究还发现FGFR3突变患者均有t（4；14）[9]。一项来自欧洲的最新研究对153例携带t（4；14）的MM患者进行测序发现，38％合并FGFR3突变，是所有突变中比例最高的，远高于其他MM中的常见突变如KRAS（11％）、NRAS、BRAF等[10]。本研究中初诊MM患者的FGFR3突变率为14.1％，t（4；14）群体55％伴FGFR3突变，明显高于前述几项早期研究，这可能与近年来测序深度增加、灵敏度及准确度提升有关。我们的数据同样略高于最新的欧洲数据，提示FGFR3突变在不同人种间可能存在异质性，因此，中国MM患者较高的FGFR3突变率有待于进一步深入研究。

FGFR3基因位于染色体4p16.3，为成纤维细胞生长因子受体家族的一员，包含19个外显子及18个内含子，主要通过与配体（FGFs）结合参与细胞内信号转导，调节细胞增殖、分化和凋亡等[11]，目前在多种恶性肿瘤中均已发现FGFR3的异常表达[12]。FGFR3最常见的突变发生于胞外和跨膜区蛋白的编码区，导致受体二聚化和不依赖于配体的信号传导增加，进一步激活下游通路[13]。在MM中，FGFR3可以通过激活下游非经典NF-κB通路、PI3K和STAT等信号通路影响多发性骨髓瘤细胞的增殖和凋亡[14]。FGFR3对MM预后的影响存在一定争议。有研究显示FGFR3高表达可促进MM进展[15]、导致更差的预后[16]，但也有研究指出FGFR3表达水平与预后无关[17]–[18]。但目前对FGFR3突变的临床价值及预后意义研究仍较少。为此，我们对198例患者的临床特征及生存进行分析，结果显示FGFR3突变患者具有更低的白蛋白水平、更高的β_2_-微球蛋白水平与R-ISS分期，提示FGFR3突变可能预示着更高的肿瘤负荷；有趣的是，我们发现FGFR3突变患者中IgA型MM的比例略高于未突变患者，既往研究也发现t（4；14）的患者具有更高的IgA型比例[16],[19]，这进一步提示了FGFR3突变与t（4；14）的重要关联。本研究生存分析显示，具有FGFR3突变的患者生存更差，进一步的Cox回归分析提示FGFR3突变是影响MM患者预后的独立危险因素。既往对其他类型肿瘤的研究也发现，FGFR3突变与肝癌的恶性程度、膀胱癌[20]的分级分期等密切相关，且FGFR3突变的膀胱癌患者更易复发，我们的研究进一步提示了FGFR3突变在MM中的预后价值，为此，需加强对FGFR3突变检测的重视，以帮助高危MM的精准识别。

对于FGFR3突变患者的治疗方案目前尚无统一认识，可参考t（4；14）患者采用新药治疗方案，含达雷妥尤单抗和蛋白酶体抑制剂的方案被证实可改善t（4；14）患者的生存[21]。尽管如此，新药时代下，t（4；14）仍是预示疾病疗效不佳、预后差的重要因素，我们的研究进一步提示FGFR3突变同样预示了不良预后，为此，更精准的靶向治疗可能是改善FGFR3突变患者预后的有效手段。目前对靶向FGFR3的单抗、抗体偶联药物、酪氨酸激酶抑制剂已在肿瘤治疗中有了初步探索，且已有产品被美国美国食品药品监督管理局（Food and Drug Administration, FDA）批准用于治疗伴FGFR3改变的尿路上皮癌[12]，靶向FGFR3在MM中的疗效也在临床前研究中得以初步证实[22]–[24]，有待于后续进一步的深入研究验证其有效性。

综上所述，本研究采用NSG技术对198例初诊MM患者进行了FGFR3基因突变的分析，是目前国内较大的针对MM中FGFR3突变的研究。我们的结果显示FGFR3突变与MM中的常见易位t（4；14）密切相关，其发生率既往可能被低估，其在MM中预示着更高的肿瘤负荷、更差的疾病预后。鉴于本研究为单中心回顾性研究，存在样本量较小、研究人群单一等问题，有待于国内更多的多中心、扩大样本量、前瞻性研究，进一步阐明FGFR3突变在MM中的临床价值。
